# The Anticancer Effect of Napabucasin (BBI608), a Natural Naphthoquinone

**DOI:** 10.3390/molecules28155678

**Published:** 2023-07-27

**Authors:** Zeyang Shao, Heng Wang, Haiyan Ren, Yinxiang Sun, Xiuping Chen

**Affiliations:** 1 State Key Laboratory of Quality Research in Chinese Medicine, Institute of Chinese Medical, University of Macau, Macao, China; mc25501@umac.mo (Z.S.); mc15347@umac.mo (H.W.); mc15376@umac.mo (H.R.); 2 Zhuhai People’s Hospital (Zhuhai Hospital Affiliated with Jinan University), Zhuhai 519000, China; 3 Department of Pharmaceutical Sciences, Faculty of Health Sciences, University of Macau, Macao, China; 4 MoE Frontiers Science Center for Precision Oncology, University of Macau, Macao, China; 5 GMU-GIBH Joint School of Life Sciences, The Guangdong-Hong Kong-Macau Joint Laboratory for Cell Fate Regulation and Disease, Guangzhou Medical University, Guangzhou 511436, China

**Keywords:** napabucasin, cancer, STAT3, NQO1

## Abstract

Napabucasin (also known as BBI608) is a natural naphthoquinone originally identified as a cancer cell stemness inhibitor. Accumulated in vitro and in vivo evidence demonstrated that napabucasin showed significant anticancer effects in various types of cancers. Napabucasin inhibits cancer cell proliferation, induces apoptosis and cell cycle arrest, and suppresses metastasis and relapse. Such anticancer activities of napabucasin mainly rely on the inhibition of cancer stemness by targeting signal transducer and activator of transcription 3 (STAT3) and its related gene inhibition. However, several novel molecular targets for napabucasin, such as NAD(P)H:quinone oxidoreductase 1 (NQO1) and thioredoxin reductase 1 (TrxR1), have been reported. Napabucasin represents a promising anticancer lead for multiple cancers. In this mini review, the anticancer potential and the molecular mechanism of napabucasin will be briefly highlighted.

## 1. Introduction

Cancer is one of the most lethal diseases in the world, with a high annual mortality rate. So far, it is the second leading cause of death in the United States [1], and it has surpassed smoking as the main cause of death in China [2]. Surgy, radiotherapy, and chemotherapy are included as the most common conventional management for most cancers. Despite the fact that novel therapeutics, such as immunotherapy, etc., have shown promising results in specific patients [3], chemotherapy, including targeted therapy, and the fight against cancer by using cytotoxic compounds, hormone drugs, biological response modifiers, etc. are the most basic clinical treatment options in many developing countries. Despite the various drugs available in the anticancer menu, their prescriptions are often limited by the side effects, and their efficacy is frequently compromised by the inevitably developed drug resistance. Therefore, the discovery of anticancer drugs with high efficacy and minimal toxicity is urgently needed.

Natural products isolated from plants, animals, minerals, and microorganisms are important sources for anticancer drug research and development. The huge number and structural diversity make them a gift from nature for lead discoveries. Quinonoid monomers that widely exist in higher and lower plant species, including naphthoquinones, phenanthrenequinones, benzoquinones, and anthraquinones, have shown favorable anticancer activities [4]. In particular, anthracyclines (adriamycin, epirubicin, daunorubicin) that have an anthraquinone backbone have been widely used for various cancers for many years [5].

Napabucasin (BBI608) is a natural naphthoquinone isolated from *Newbouldia laevis*, *Ekmanianthe longiflora*, and *Handroanthus impetiginosus.* It is an orally available small molecule with wide-spectrum anticancer activities and is now in clinical trials for cancer treatment [6]. Napabucasin was discovered and originally identified to be a cancer stemness inhibitor by suppressing the signal transducer and activator of transcription 3 (STAT3) [7], whose phosphorylation till pSTAT3 triggers the transcription of genes implicated in promoting tumor growth and survival and in regulating inflammation in the tumor microenvironment. Recently, it was found to be a NAD(P)H:quinone oxidoreductase 1 (NQO1) substrate and functioned as an NQO1 bioactivatable drug [8]. In this review, we summarize the up-to-date works on the anticancer efficacy and mechanism of napabucasin.

## 2. Anticancer Activity of Napabucasin

Napabucasin showed multiple anticancer activities, including proliferation inhibition, cell death induction, cell cycle arrest, metastasis suppression, drug resistance overcoming, and stemness inhibition, etc., which were documented in many benchworks.

### 2.1. Cell Proliferation Inhibition and Cell Death Induction

As reported and summarized in Table 1, napabucasin inhibits cancer cell proliferation and growth of myeloid leukemia, liver, lung, ovarian, breast, B-cell lymphoma, osteosarcoma, glioblastoma, colorectal, prostate, biliary tract, pancreatic ductal, and hypopharyngeal cancer cells. Differential cytotoxicity of napabucasin was detected and observed after 6 h of treating different cell lines with increasing BBI608 concentrations [8]. In those cancer cell lines, the IC50s of napabucasin were typically at the μM or even nM level, which is substantially lower than the IC50s of several widely studied natural substances. In hepatocellular carcinoma, for example, napabucasin was far more active than cryptotanshinone in terms of anticancer activity [9].

Inducing cancer cell apoptosis is one of the general anticancer bases for napabucasin, and accumulated studies have demonstrated that napabucasin could activate intrinsic and extrinsic apoptosis pathways to cause apoptotic cell death. The cell viability of NHL (non-Hodgkin’s lymphoma) cell lines decreased with increasing concentrations of napabucasin, ranging from 0.001 to 2.0 μM for 48 h and 72 h, and, in DLBCL (diffuse large B-cell lymphoma) cells treated with napabucasin, the expression of cleaved caspase-3 and cleaved PARP significantly elevated in a concentration-dependent manner [10,11]. In addition, in drug-resistant lung cancer cells, napabucasin significantly induced cell apoptotic death when used as a single therapeutic agent [12]. In cisplatin-resistant small cell lung cancer (SCLC), napabucasin induces apoptosis by cleavage PARP and suppression of antiapoptotic proteins Mcl-1 and survivin [13].

**Table 1:** The anticancer properties of napabucasin in vitro.

Cell	Pathology	Treatment	Result	Ref.
HuCCt-1, NOZ	Intrahepatic cholangiocarcinoma	2 μM for 24 h	Suppresses cancer stemness	[14]
BxPC-3	Pancreatic adenocarcinoma	2.5 μM for 1 h	Resensitizaion of cells to radiation therapy (RT) and chemoradiotherapy (CRT)	[15]
U87MG, LN229	Glioblastoma	5 μM for 48 h	Induces cell cycle arrest, cell apoptosis; inhibits STAT3, cell migration, and invasion	[16]
143B, MG63, U2OS, KHOS	Osteosarcoma	5 μM for 72 h	Induces cell apoptosis; inhibits STAT3	[11]
HCT116, HT29	Colorectal carcinoma	1 μM for 36 h	Induces ROS generation, DNA damage, suppresses angiogenesis	[17]
Huh7, HepG2, murine Hepa1-6 cells, HepG2.2.15	Hepatoma	2 μM for 48 h	Induces cell apoptosis, cell cycle arrest; suppresses cancer stemness	[9]
H460, H1299, SKMES-1	Lung carcinoma	1 μM for 72 h	Induces cell apoptosis; suppresses cancer stemness; resensates cisplatin resistance cells	[12]
PC-3, 22RV1	Prostatic carcinoma	1 μM for 2 to 5 days	Induces cell apoptosis, cell cycle arrest; suppresses cancer stemness	[18]
AML	Acute myeloid leukemia	Designed dose for 24 h	Induces cell cycle arrest, DNA damage, cell apoptosis; inhibits STAT3	[19]
A2780, SKOV3, ID8	Ovarian carcinoma	2 μM for 12 h	Induces apoptosis; resensitization of cell to tamoxifen or paclitaxel; inhibits STAT3, cell migration, and invasion	[20]
FaDu	Pharyngeal squamous cell carcinoma	2 μM for 24 h	Suppresses cancer stemness	[21]
DLD1, HCT116	Colorectal carcinoma	2 μM for 24 h	Blocked cell survival and self-renewal	[21]
Melanoma (ret melanoma cell)	Melanoma	1 μM for 72 h	Inhibits cell proliferation	[22]

### 2.2. Disruption of Cell Cycle

It is frequently maintained that the majority of cancer cells move through the cell cycle in an uncontrollable manner. Because of mutations that permit cell cycle advancement and impede exit, cancer cells keep dividing [23]. As a result of cancer cells’ higher reliance on cell cycle control regulatory pathways, there are opportunities to focus on disturbing the cell cycle, which represents a novel strategy for anticancer drug development. Abemaciclib, palbociclib, and ribociclib were approved by the FDA for breast cancer treatment as functioning as the CDK4/6 inhibitors that arrest the cell cycle in G1-phase [24]. Napabucasin owns such an ability as well, and it has been well documented in many experiments that napabucasin is a potent cell cycle arrest inducer. Of note, different from those singly disrupting one phase in a whole cell cycle, napabucasin could trigger cell cycle arrest in different cycle phases in cancer cells, dependent on the cancer type. For example, napabucasin attenuated the proliferation of glioma cancer cells by inducing G1/S-phase arrest in U87MG and LN229, along with its disruption of the cell cycle in CT26 colorectal cancer cells, and PC-3 and 22RV1 prostate cancer cells [16,18,25]. While in HGC-27 human gastric cancer cells, MDA-MB-231 breast cancer cells, and HeLa cervical cancer cells, napabucasin induces S-phase arrest [26] and induces G2/M phase arrest in HepG2.2.15 hepatic carcinoma cells [9]. Consistent with its ability to perform in breast cancer cells, after 24 h of dosing, napabucasin caused the cell cycle of H146 and H446 cells to arrest in the S-phase [13]. Accumulation in the percentage of cycling Molm13 acute myeloid leukemia cells in the G0/G1 phase and a significant reduction in the number of cells cycling in the S-phase were observed clearly after administration of napabucasin at increasing dosages [19]. Such alteration of the proportion between those two phases was detected, as well, in the U87MG cell line and LN229 cell line with increasingly introducing napabucasin [16]. This G1 phase arrest induced by napabucasin might be mediated by the upregulation of P21 and a reduction in Cyclin D1 and CDK4 in Molm13 cells and U87MG and LN229 cells [16,19].

### 2.3. Suppress Metastasis and Improve Drug Resistance

One of the main causes of cancer patients’ mortality is tumor metastasis, which accounts for about 90% of cancer-related deaths. The process of metastasis is the migration of cancer cells from their originating site to gradually colonized distant organs [20]. From the results of wound healing assays, transwell migration, and invasion assays on EOC20 cell lines after 24 h of the administration of napabucasin, fewer migrating SKOV3 ovarian cancer cells and A2780 ovarian cancer cells began to migrate as the drug concentration increased [27]. Additionally, better than ReoT3D and CPT-11 in monotherapy, napabucasin led to more antimigration action on CT26 mouse colon cancer cells [25]. Similar inhibited migration and invasion were also observed in U87MGc cells and LN229 cells and possibly by suppressing matrix metalloproteinase-2 (MMP2) and MMP9 [16], which could decompose the extracellular matrix (ECM) and stimulate cell invasion and metastasis through epithelial–mesenchymal transition (EMT) in vitro and in vivo. However, the detailed mechanisms remain unclear.

The effect of napabucasin on drug resistance is mentioned in a few studies. In nonsmall cell lung cancer (NSCLC) cells, napabucasin not only inhibits cell proliferation of cisplatin-resistant NSCLC cells alone but also enhances the cytotoxicity of cisplatin in drug-resistant sublines. As an independent treatment, napabucasin dramatically reduced the ability of all cisplatin-resistant sublines to proliferate in comparison to untreated cells. Napabucasin dramatically reduced cell proliferation in all resistant NSCLC sublines that represented each histological subtype of this cancer type when combined with cisplatin [12]. This finding suggests that napabucasin should be researched further as a novel drug for resensitizing NSCLC cells to cisplatin’s cytotoxic effects. Besides those resistant to cisplatin, napabucasin stimulates ovarian cancer cell death by enhancing its sensitivity to paclitaxel both in vitro and in vivo [27].

### 2.4. Inhibition on Cancer Stemness

Cancer stem cells (CSCs), also known as tumor-initiating cells, are a small subset of cells within a heterogeneous tumor that have the unique ability to initiate new cancer upon transplantation and share characteristics with embryonic and stromal stem cells, such as the ability to self-renew (divide to make more stem cells without transforming into a specialized cell) and differentiate (transform into a specialized cell) [28]. Such CSCs are usually considered the key factor in cancer development, relapse, drug resistance, and metastasis [21,29]. Napabucasin was originally demonstrated to inhibit cancer stem cell proliferation and stemness properties as evidenced by the inhibition of sphere formation and clonogenic growth in malignant gliomas cancer cells, hepatic cancer cells, prostate cancer cells, and biliary tract cancer cells [9,14,16,18,30]. In PCa stem cell stemness, napabucasin treatment dropped the protein expression level of Nanog, Klf4, survivin, C-myc, and β-catenin in PrCSCs in a dose-dependent manner. The considerably decreased mRNA expression of Nanog, Klf4, survivin, and β-catenin in napabucasin treatment was observed through qRT-PCR [18]. Such decreased levels of the stemness marker indicate the inhibitive effect of napabucasin on cell stemness. More importantly, although well performed in cancer cells, napabucasin did not appear to have adverse effects on hematopoietic or other normal adult stem cells. Besides these, the drug-resistant cancer cells showed stronger stemness properties, such as higher sporogenesis ability, colony formation ability, aldehyde dehydrogenase activity, and expression of stemness key factor [31]. One example is in MCF7/MCF7-R breast cancer cells with tamoxifen resistance. Behaving as the canonical STAT3 inhibitor, napabucasin attenuated the stemness of breast cancer stemness [32] and another type of cancer [30] and decreased stemness marker expression, colony formation ability, and aldehyde dehydrogenase (ALDH) activity of MCF7-R, which exhibited a higher stemness to MCF7 cells. As such, napabucasin reverses the tamoxifen resistance of MCF7/MCF7-R breast cancer cells and enhances its sensitivity toward tamoxifen [32].

Several signal pathways are involved in the regulation of cancer stem cell proliferation and differentiation, such as the STAT3, Wnt, and PI3K/Akt pathways [22]. Napabucasin inhibits cancer stemness mainly by inhibiting STAT3 and its target genes. Napabucasin kills cancer stem cells and decreases stem-cell-related biomarkers Nanog, SOX2, Oct4, CD90, Klf4, survivin, c-Myc, and β-catenin in liver cancer [9], breast cancer [32], prostate cancer [18], and so on. Napabucasin blocked the survival and self-renewal of cancer stem cells and inhibited their related proteins and gene expression in pharynx squamous cell carcinoma FaDu cells. In contrast, some regular chemical drugs or kinase-targeted drugs induced cancer stem-cell-related gene expression [30].

### 2.5. In Vivo Evidence

The anticancer effect of napabucasin has been confirmed in various animal models. As a monotherapy, napabucasin administration reduced tumor volume and inhibited tumor growth in an orthotopic tibial osteosarcoma model, an orthotopic glioma model, an HCC homograft mice model, and human acute myeloid leukemia xenograft murine models [9,11,16,19]. It also significantly prolonged the life span of tumor-burden mice in glioma and melanoma mice models [16,33]. Furthermore, napabucasin treatment alleviated bone osteolysis and bone destruction in a 4-week-old female BABL/c nude mice model [11]. In addition, napabucasin administration inhibited metastasis in an osteosarcoma nude mice model, a colon cancer cells liver metastasis model, and a pancreatic cancer xenograft model [11,30].

The in vivo anticancer effect of napabucasin mainly depends on the inhibition of stem cells, cell proliferation, and induction of apoptosis. Napabucasin downregulates stemness-related genes Nanog, SOX2, Klf4, and Oct4 CD90 and EpCAM mRNA expression levels both in tumor cells and tumor tissues [9]. Napabucasin treatment decreases Ki67-positive cells and increases apoptotic cells, suggesting that napabucasin inhibits cancer cell proliferation and triggers apoptosis [9,10].

Combination therapy of napabucasin with other drugs shows a synergistic anticancer effect. Napabucasin synergized with doxorubicin, docetaxel, and paclitaxel in a DLBCL diffuse large B-cell lymphoma xenograft model [10], prostate cancer xenograft models [18], and a model of peritoneal tumor of ovarian cancer [27], respectively. The undergoing mechanism of its synergistic potential could be its inhibition of cancer cell stemness or enhancement of certain anticancer medications, simultaneously performing its apoptosis-inducing ability. The reported anticancer effect of napabucasin in vivo is summarized in Table 2.

### 2.6. Molecular Targets

STAT3, an oncogenic transcription factor, is a well-known promising anticancer target [36]. It has been verified as being essential in controlling the antitumor immune response because it serves as a point of convergence for many oncogenic signaling pathways. It is widely hyperactivated in both cancer and noncancerous cells within the tumor ecosystem and plays significant roles in inhibiting the expression of essential immune activation regulators and promoting the production of immunosuppressive factors. Among STAT protein family members, STAT3 is involved in numerous biological processes, including cell proliferation, survival, differentiation, and angiogenesis. Several inhibitors and their analogs of STAT3 have been well testified as effective anticancer drugs (some natural STAT3 inhibitors are listed in Table 3), such as Rapamycin, Angoline, Shikonin, and so on [37,38,39]. As mentioned above, the inhibitory effect of napabucasin on stemness is mediated by STAT3 [16]. With this, napabucasin is enabled to perform those properties listed above.

However, recent evidence showed that NQO1, a two-electron reductase involved in the detoxification of quinones and the bioactivation of certain quinones [40], was a major determinant of napabucasin’s efficacy. In those solid tumors with high expression of NQO1, such as lung cancer, and ovarian cancer, NQO1 was regarded as the therapeutic target due to its promotion of cancer cell growth at the early stage of carcinogenesis by binding and stabilizing the mutant or wild-type p53 and then inhibiting its degradation [41]. Napabucasin is an NQO1 substrate and selectively kills NQO1 high-expression cancer cells. In particular, the inhibitory effect of napabucasin on STAT3 was found to be mediated by NQO1-derived reactive oxygen species (ROS) [8]. It is reasonable that NQO1 acts as a target for napabucasin from a chemical point of view. Napabucasin has a naphthoquinone structure, and many compounds with this structure, such as β-lapachone and 2-methoxy-6-acetyl-7-methyljuglone (MAM), have been found to exert antitumor activity by targeting NQO1 [41,42]. Thus, napabucasin might act as an NQO1 bioactivatable drug. Consistent with this is the excessive ROS generation induced by napabucasin in NQO1 high-expression cells [26]. However, for those cells with low expression of NQO1, napabucasin treatment still caused the accumulation of ROS, which suggests that other targets and mechanisms should be involved in this process. Indeed, although napabucasin binds cytochrome P450 oxidoreductase with a weaker affinity than that of NQO1, once binding, it could also induce ROS generation [8], and the subsequent events occurred. As NQO1 activation triggers nonapoptotic cell death termed noptosis [43], the cell death induced by napabucasin may be cell-type-dependent. With this idea, as mentioned above, the anticancer activity on disrupting the cell cycle differing in cancer cells from type to type probably depends on different CDK targets or others.

**Figure 1:**
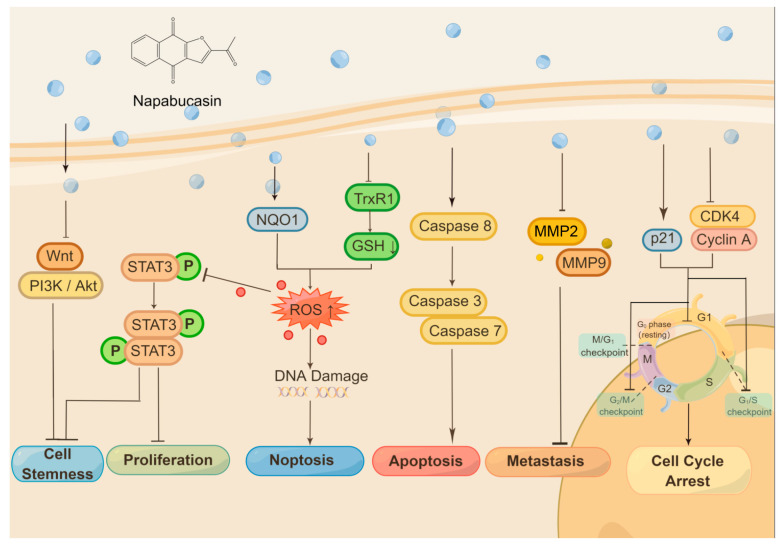
The anticancer molecular mechanisms of napabucasin.

Besides STAT3 and NQO1, napabucasin is identified to be an inhibitor of thioredoxin reductase 1 (TrxR1). It acts as a redox cycling substrate of TrxR1 and irreversibly inhibits cellular TrxR1 activity as a few STAT3 inhibitors [44]. Recently, aldehyde dehydrogenase was proposed to be another napabucasin target using thermal proteome profiling in whole zebrafish embryo lysate [45]. The anticancer molecular mechanisms of napabucasin are summarized in Figure 1.

Napabucasin shows great anticancer activity via different molecular mechanisms. Napabucasin itself inhibits several signaling pathways, such as Wnt, PI3K/Akt, and STAT3, and stands the inhibition of cancer cell stemness as one consequence, another as inhibition of cancer cell proliferation. Inhibition of STAT3 through ROS generation is mediated by NQO1, as napabucasin is an NQO1 substrate. Napabucasin is also identified as a TrxR1 inhibitor. The activation of the caspase 3/7/8 induced by napabucasin drives the cancer cell to apoptosis. In contrast, ROS generation triggers both STAT3 inhibition and DNA damage and finally induces noptosis, a nonapoptotic cell death. Meanwhile, napabucasin inhibits MMP2/MMP9, which takes the suppression of cancer metastasis as a result. In addition, napabucasin can disrupt the cell cycle on different phases or checkpoints in cancer cells from type to type. Figure 1 was drawn by Figdraw (ID:UIISU46533).

## 3. Clinical Trials of Napabucasin

As a potential anticancer drug, napabucasin has shown preclinical efficacy in anticancer studies ranging from monotherapy to combination with conventional chemotherapy agents. Positive safety profile results in phase I have encouraged further testing of napabucasin as a monotherapy [46] and in combination with conventional therapies in phase II and III studies [47] for anticancer treatment.

The positive tolerability and antitumor activity of napabucasin in many cancers were well testified and verified in phase I/II studies, and its combination with standard therapy is ongoing. Obtained from patients with AML ex vivo, data showed that napabucasin is highly active against diverse AML cell lines in the primary patient sample with ages from 21 to 83, and the younger patients (<60 years old) had a median EC50 of 4.52 ± 4.26 μmol/L compared to older people (≥60 years old) with 6.84 ± 3.66 μmol/L [19]. A phase Ib/II study demonstrated that napabucasin at 480 mg BID can be safely combined with temozolomide (TMZ) at full dose, and the disease control rate was observed in five patients (55.5%), of whom four achieved PR (44.4%), and one achieved SD (11.1%), showing encouraging antitumor activity in patients with recurrent glioblastoma [48]. Not only with TMZ, napabucasin can also be safely combined with weekly paclitaxel (NCT01325441) with acceptable tolerability in patients with heavily pretreated platinum-resistant ovarian cancer (PROC), and in 56 patients, DCR was 48%, ORR was 18%, median progression-free survival was 15 weeks, and median overall survival was 38 weeks [34]. This combination showed promising antitumor effects for many types of cancer, especially in gastric cancer and GEJ adenocarcinoma [34]. In trial NCT02231723, the disease control (PR + SD) of 27 evaluable patients (out of 29, 93%) was observed, with 23 patients (79.3%) showing tumor regression, out of whom 10 patients (34.5%) achieved PR (RECIST 1.1: 33–78% regression). Among 37 intent-to-treat patients, disease control (PR + SD) was observed in 27 patients (73%), with tumor regression observed in 23 patients (62.2%), out of whom 10 patients achieved PR (27%) [35]. Moreover, in the therapy of colorectal cancer (NCT02753127) with a combination of napabucasin and FOLFIRI +/− Bev., the general study population had an OS improvement from 12.54 to 15.68 months [49].

All clinical trials shared similar PK and TEAEs with the reported results of napabucasin. However, one CanStem303C phase III research (NCT02753127) failed to fulfill either of its major endpoints; in patients with mCRC, treating napabucasin with FOLFIRI (with or without bevacizumab) did not enhance OS in either the overall study population or the biomarker-positive subgroup. Even in a few trials, the interpretation of the efficacy data was limited by the small number of patients (NCT02358395). As such, more effective clinical trials need to be urgently conducted.

## 4. Side Effects and Toxicity of Napabucasin

With awareness of the promising anticancer abilities of napabucasin, it is of need to testify to its safety to guide clinical medication. Napabucasin is a targeted cancer drug that was projected to be less hazardous to patients than standard chemotherapy due to its specificity of action, which targets cancer cells rather than normal cells [30]. In preclinical research, napabucasin was well tolerated and showed a few symptoms of toxicity on hematopoietic stem cells or other normal adult stem cells in toxicology assessments [50]. In addition, during treatment and post-treatment surveillance, in animal experiments, rats given napabucasin showed no changes in body weight [50]. However, in several clinical trials, treatment-emergent adverse events (TEAEs) happened. The most common TEAEs were grade 1–2 nausea, diarrhea, anorexia, and fatigue, which occurred in a phase I dose-escalation study conducted on adult patients with advanced cancer within oral administration of napabucasin for 4 weeks [46]. Because of the differences in the treatment method, grade 3 TEAEs occurred in 73.8% of patients treated with napabucasin and FOLFIRI, and gastrointestinal disorder syndrome happened in over 41.0% of this group [47]. Considering patients’ differences, eight healthy male subjects were treated with napabucasin and suffered from, at the least, gastrointestinal disorder events, which were mostly observed [51]. Interestingly, diarrhea started, as reported, at approximately 4.5–5.0 h after administration and lasted up to 11 days. Since the intake of napabucasin was turned off, all disorder incidents were resolved in this experiment. However, the reason for inducing this side effect of napabucasin still stays unknown. As such, it is generally recommended that patients with gastrointestinal-related AEs could receive up-front management with antidiarrheal agents and combination therapy, if necessary, and the trial conductor should consider it important to monitor patients for such side effects typical during the first cycle of the treatment, especially in older patients and those at risk for complications [52].

Notwithstanding the previously documented clinical gastrointestinal side effect without too much severity, napabucasin was found to play a severe role in the skeleton system of mice. STAT3 essentially regulates bone anabolism in rodent species [53]. Notably, napabucasin was found as an inhibitor of the process of osteogenic differentiation when treating napabucasin on bone marrow mesenchymal stem cells (BMSCs) [54]. Being the base and parallel to this finding, the inhibition of the expression of STAT3 could disrupt the bone formation process in osteoblasts [55]. Napabucasin modulated Ocn transcription through STAT3 and eventually affected the osteogenic differentiation of BMSCs. The result of HE staining on the femora found that there was bone loss in the napabucasin injection group compared with the group without napabucasin [54]. All those works indicate the induction of napabucasin toward impaired bone formation and, consequently, bone loss. However, with such a discovery, prostate cancer treated with napabucasin successfully prevented bone metastatic progression by inhibiting STAT3 activity [56].

## 5. Concluding Remarks

The immense chemical diversity and favorable properties endow natural products as a major resource for drug discovery [57]. The anticancer potentials are of particular interest in the pharmacological screening and evaluation of natural products. Though thousands of natural products have been claimed to show anticancer effects in experimental models each year [58], only a few enter clinical trials. Among them, napabucasin shows significant translational potential. By retrieving https://clinicaltrials.gov/ (accessed on 3 January 2023), 27 clinical trials are ongoing for the evaluation of the safety, tolerance, pharmacokinetics, and anticancer efficacy of napabucasin, which has been well summarized recently [31].

In the era of targeted therapy and precision medicine, a unique target or mechanism of action favors innovative drug discovery. As the first-in-class cancer stemness inhibitor, napabucasin represents a direction and strategy for fighting cancer. However, it shows a spectrum of anticancer activity, suggesting other mechanisms besides stemness inhibition. NQO1, the phase II enzyme that is highly expressed in various cancers, is a promising novel anticancer drug target for NQO1-positive cancers. In particular, a 100-fold increase in NQO1 expression was found in pancreatic cancer [59], the most lethal cancer type with a 5-year overall survival rate below 10% [60]. β-Lapachone, an NQO1 bioactivatable drug, is under clinical trial for pancreatic cancer treatment. Given that NQO1 might be the primary target of napabucasin, the NQO1 expression level should be considered for clinical design and data analysis.

Combination therapies offer attractive chances for better efficacy and reduced toxicity for chemotherapeutic drugs [61]. Clinical trials of napabucasin in combination with paclitaxel, Nab-paclitaxel, sorafenib, temozolomide, etc., have been launched. However, we need more solid benchworks to support the rationales for these combinations, as only a small number of publications touch this. After all, “synergistic combination” is easier said than done from a pharmacological aspect.

## Figures and Tables

**Table 2:** The anticancer properties of napabucasin in vivo.

Model	Treatment	Result	Ref.
U87MG intracranially xenotransplanted in nude mice	40 mg/kg, i.p. every other day for 2 months	Suppresses tumor growth with prolonged survival rate than vehicle	[16]
143B injected into the right tibial medullary cavity of BABL/c nude mice	10 or 20 mg/kg, i.p. every 3 days within eight injections	Decreases tumor volume; inhibits osteosarcoma lung metastasis	[11]
SUDHL-6 subcutaneously injected into the flank of NOD/SCID mice	50 mg/kg, p.o. daily; doxorubicin 3 mg/kg, i.v, once weekly; combination treatment for 15 days	Suppresses tumor growth without obvious organ injury	[10]
Hepa1-6 subcutaneously injected into the left axilla of C57BL/6J male mice	20 mg/kg, intraperitoneal injections every 2 days within eight injections	Decreases tumor volume; increases proportion of apoptotic tumor cells	[9]
PC-3 or 22RV1 subcutaneously injected into dorsal flank in nude mouse	40 mg/kg, i.p. every 3 days	Suppresses tumor growth; inhibits spherogenesis	[18]
Molm-13 injected into NOD-Prkdc^−/−^IL2rg^−/−^ (NPI) mice after one Gy irradiation	60 mg/kg, p.o. for 2 weeks	Decreases tumor volume	[19]
Model A: SKOV3 subcutaneously injected into BALB/c mice; Model B: ID8 intraperitoneally inoculated into C57BL/6 mice	Model A: 40 mg/kg, i.g. daily for 21 days; Model B: 40 mg/kg, p.o. for 6 days; paclitaxel 12 mg/kg, i.p. once; combination treatment daily for 6 days	Decreases tumor volume and weight without noticeable ascites and toxicity Inhibits pSTAT3-positive cells, STAT3 activity, and Ki67 expression	[20]
Model A: PaCa-2 inoculated s.c. in athymic nude mice Model B: HT29 injected under spleen capsule of nude mice	Model A: 20 mg/kg, i.p. every 3 days for 4 weeks; Model B: 20 mg/kg, i.p. 5 days per week for 4 weeks	Inhibits cancer relapse, metastasis, and stemness Suppresses tumor growth	[21]
CT26 subcutaneously injected into the right flanks of BALB/c mice	50 mg/kg of derivatives 8q, p.o. daily for 4 weeks	Suppresses tumor growth; decreases tumor volume	[34]
MDA-MB-231 subcutaneously injected into BALB/c nude mice	5 or 10 mg/kg once daily of derivatives A11, i.p., for 21 days	Decreases tumor volume and weight; inhibits p-STAT3	[35]

**Table 3:** Natural STAT3 inhibitors with anticancer purposes.

Inhibitor	Origin	Treatment of Cancer
Curcumin	*Curcuma longa* L. (*turmeric*)	Human prostate cancer (LNCa, C4-2B), human breast cancer (MDA-MB-231), colon carcinoma (C-26), human ovarian adenocarcinoma (SKV3), etc.
Cucurbitacin II	*Cucumis sativus* (*cucumber*) and *Cucumis melo* L. (*melon*), etc.	Human breast cancer (MDA-MB-231), liver cancer (HepG2), human lung cancer (A549), etc.
Honokiol	*Officinalis, obovata* and *grandiflora*	Human lung cancer (A549), liver cancer (HepG2), human skin cancer, etc.
Guggulsterone	*Commiphora mukul*	Pancreatic cancer, hepatocellular carcinoma, head and neck squamous cell carcinoma, cholangiocarcinoma, etc.
Resveratrol	Grapeskin, blueberries, raspberries, mulberries, and peanuts	Human breast cancer (MDA-MB-231, MDA-MB-453, MDA-MB-468), pancreatic cancer (Panc-1, Colo-357), prostate cancer (LNCaP, DU145), etc.
Berbamine	*Berberis amurensis*	Hepatocellular carcinoma, pancreatic cancer
Flavopiridol	*Aphanamixis polystachya*	Human chronic lymphocytic leukemia cells, human squamous cell, prostate carcinoma, etc.

## Data Availability

Not applicable.
